# Pathogen prevalence and abundance in honey bee colonies involved in almond pollination

**DOI:** 10.1007/s13592-015-0395-5

**Published:** 2015-10-21

**Authors:** Ian Cavigli, Katie F. Daughenbaugh, Madison Martin, Michael Lerch, Katie Banner, Emma Garcia, Laura M. Brutscher, Michelle L. Flenniken

**Affiliations:** 1 Department of Plant Sciences and Plant Pathology, Montana State University, Bozeman, MT 59717 USA; 2 Institute on Ecosystems, Montana State University, Bozeman, MT 59717 USA; 3 Department of Microbiology and Immunology, Montana State University, Bozeman, MT 59717 USA; 4 Department of Mathematical Sciences, Montana State University, Bozeman, MT 59717 USA

**Keywords:** honey bee colony health, honey bee viruses, Black queen cell virus, Lake Sinai virus, almond pollination

## Abstract

Honey bees are important pollinators of agricultural crops. Since 2006, US beekeepers have experienced high annual honey bee colony losses, which may be attributed to multiple abiotic and biotic factors, including pathogens. However, the relative importance of these factors has not been fully elucidated. To identify the most prevalent pathogens and investigate the relationship between colony strength and health, we assessed pathogen occurrence, prevalence, and abundance in Western US honey bee colonies involved in almond pollination. The most prevalent pathogens were Black queen cell virus (BQCV), Lake Sinai virus 2 (LSV2), Sacbrood virus (SBV), *Nosema ceranae*, and trypanosomatids. Our results indicated that pathogen prevalence and abundance were associated with both sampling date and beekeeping operation, that prevalence was highest in honey bee samples obtained immediately after almond pollination, and that weak colonies had a greater mean pathogen prevalence than strong colonies.

**Electronic supplementary material:**

The online version of this article (doi:10.1007/s13592-015-0395-5) contains supplementary material, which is available to authorized users.

## Introduction

Insects pollinate agricultural crops and plant species that enhance landscape biodiversity. The value of insect pollination worldwide is $153 billion annually, and honey bees (*Apis mellifera*) are the primary pollinators of US crops valued at over $17 billion per year (Calderone 2012; Gallai et al. 2009). A striking example of the role of honey bee pollinators in agriculture is California almond production, where ~80 % of the world’s almonds are produced (CAB 2014). Honey bees are essential to almond production, and over 60 % of the US commercially managed honey bee colonies are involved in almond pollination from late February to early March. In 2013–2014, approximately 1.6 million bee colonies participated in this single pollination event.

Honey bee-mediated pollination is important for agricultural production. Thus, high annual US honey bee colony losses (averaging 33 % since 2006) concern growers, beekeepers, scientists, and policy makers (Spleen et al. 2013; Steinhauer et al. 2014; van Engelsdorp et al. 2007, 2008, 2009, 2012). Although the total number of US commercially managed colonies has remained at around 2.5 million since the late 1990s, beekeepers must split colonies (i.e., make two colonies from one colony) more frequently in order to make up for higher annual losses (USDA NASS). Typically, the majority of colony losses occur during the winter months and thus coincide with the almond pollination season, though a recent US management survey indicated that 2014 summer losses were also significant (~27.4 %) (Steinhauer et al. 2015). Research to date suggests that multiple biotic and abiotic factors contribute to colony health and survival (e.g., viruses, mites, microbes, bee genetics, weather, forage quality and availability, management practices, and agrochemical exposure) (Calderone 2012; Cornman et al. 2012; Ellis et al. 2010; Gallai et al. 2009; Nazzi and Pennacchio 2014; Pettis and Delaplane 2010; van der Zee et al. 2012, 2014; van Engelsdorp et al. 2009, 2012). Although no single factor is responsible for colony losses or colony collapse disorder (CCD), honey bee samples from CCD-affected colonies had greater pathogen (e.g., viruses and *Nosema*) prevalence and abundance compared to unaffected colonies (Cornman et al. 2012; Cox-Foster et al. 2007; Johnson et al. 2009; Steinhauer et al. 2015; van Engelsdorp et al. 2009). In addition, several epidemiologic and temporal monitoring studies have partially attributed colony losses to pathogens (i.e., viruses, bacteria, fungi, trypanosomatids, and mites) (Chen et al. 2014; Cornman et al. 2012; Cox-Foster et al. 2007; Evans and Schwarz 2011; Genersch and Aubert 2010; Locke et al. 2014; McMenamin and Genersch 2015; Nazzi and Pennacchio 2014; Nielsen et al. 2008; Ravoet et al. 2013; Tentcheva et al. 2004; van Engelsdorp et al. 2009, 2012). While these studies are informative, the roles of pathogens in colony mortality and the relationships between colony strength and pathogen prevalence and abundance have not been fully elucidated.

The majority of honey bee-infecting pathogens are RNA viruses, including Acute bee paralysis virus, Black queen cell virus, Israeli acute paralysis virus, Kashmir bee virus, Deformed wing virus, Sacbrood virus, Chronic bee paralysis virus (reviewed in (Brutscher et al. 2015; Chen and Siede 2007; de Miranda et al. 2013; Evans and Schwarz 2011; Genersch and Aubert 2010; McMenamin and Genersch 2015), and the Lake Sinai viruses (Cornman et al. 2012; Daughenbaugh et al. 2015; Granberg et al. 2013; Ravoet et al. 2013, 2015; Runckel et al. 2011). Longitudinal monitoring of colony health and pathogen prevalence and abundance is critical to determining the role of pathogens in colony losses, yet there have been very few studies of this nature performed in the USA.

To better understand the role of pathogens on honey bee colony health and mortality, we monitored colony health (using colony size as a proxy), pathogen prevalence, and pathogen abundance, before, during, and after the 2013–2014 almond pollination season (Delaplane and van der Steen 2013; OSU 2011). We utilized polymerase chain reaction (PCR) to test for 16 common honey bee pathogens. The majority of these pathogens were viruses including Lake Sinai virus 1 (LSV1), LSV2, LSV3, LSV4, LSV5, Black queen cell virus (BQCV), Deformed wing virus (DWV), Sacbrood virus (SBV), Acute bee paralysis virus (ABPV), Chronic bee paralysis virus (CBPV), Israeli acute paralysis virus (IAPV), and Kashmir bee virus (KBV). In addition, we tested for bacterial pathogens (i.e., *Paenibacillus larvae* and *Melissococcus plutonius*) and eukaryotic parasites including the microsporidia *Nosema* spp. and trypanosomatids (i.e., *Crithidia mellificae SF*/*Lotmaria passim*). Herein, we present data from an observational cohort study at the colony level that provides current information regarding pathogen prevalence and abundance in Western US commercially managed honey bee colonies involved in almond pollination. Enhanced understanding of the dynamics of pathogenic infections in bee colonies across different geographies and in independent beekeeping operations will inform ongoing and future epidemiologic studies aimed at investigating the potential relationships between colony health and pathogen occurrence, prevalence, and abundance on a larger scale (van Engelsdorp et al. 2013). Understanding the role of pathogens and other factors on honey bee health is critical to the development of pollinator management and conservation strategies that limit annual bee colony mortality (Gallant et al. 2014).

## Materials and methods

Additional details regarding methods are available in the Supplemental Information (Online Resource 1).

### Longitudinal monitoring and sampling of commercially managed honey bee colonies

**Figure 1.**
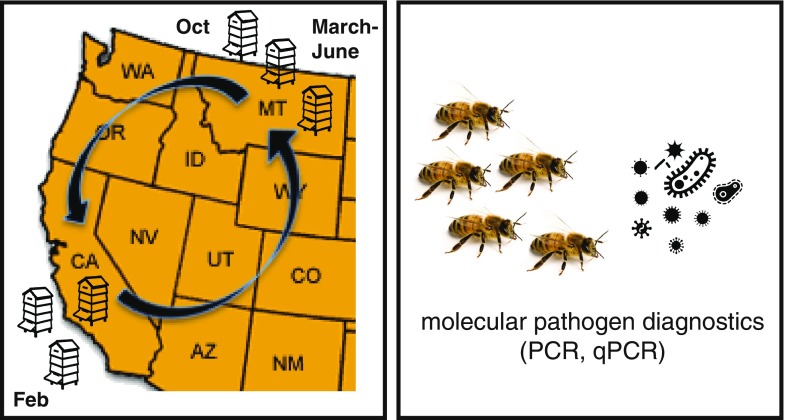
Longitudinal monitoring of commercially managed honey bee colonies, before, during, and after the 2014 almond pollination season. Honey bee colonies (*n* = 54) from three Montana-based commercial beekeeping operations were monitored before (October–November 2013), during (February 2014), and after (March–April); after 2 (June) almond pollination in California. Colony strength was measured at each sampling event. PCR was utilized to detect pathogens associated with each sample, and qPCR was utilized to determine the abundance of pathogens associated with a subset of samples (Supplemental Table S2).

Three Montana-based (Broadwater, Yellowstone, and Treasure counties) commercial beekeeping operations that transport their honey bee colonies ~1,200 miles to California (Merced and Stanislaus counties) each winter for the almond bloom provided honey bee samples before (October–December 2013), during (February 2014), and after (March/April and June 2014) almond pollination (Figure 1 and Supplemental Table S2). Colony health, using colony population size as a proxy, was assessed by the number of frames covered with honey bees (frame counts) at each sampling event (Delaplane and van der Steen 2013; OSU 2011). Colony strength was defined as follows: weak colonies (<5 frames covered with bees), average colonies (6–8 frames covered with bees), and strong colonies (>9 frames covered with bees). Live honey bee samples (~100 per sample) were obtained from the top of the frames in the middle of the colony. Samples were composed of female bees of mixed age, including nurse, worker, and forager bees. The samples were collected on ice or dry ice, stored at −20 °C, shipped on dry ice, and transferred to −80 °C prior to analysis. At the onset of the study in November 2013, each beekeeper identified 15–20 colonies of differential health. Specifically, operation 3 initiated the study with five weak, five average, and five strong colonies and provided samples at three time points; operation 2 initiated the study with five weak, 13 average, and two strong colonies and provided samples at four time points; and operation 1 initiated the study with five weak, four average, and ten strong colonies and provided samples at four time points (Supplemental Table S2). A total of 176 honey bee samples with corresponding colony strength observations were obtained and analyzed, four observations of colony strength lacked corresponding samples, and eight of the original colonies died during the course of this study (Supplemental Table S2).

### Honey bee samples

Five female bees from each sample were used for RNA extraction, cDNA synthesis, pathogen-specific PCR, and qPCR. The objective for pathogen screening was to identify the most prevalent pathogens associated with honey bees sampled from individual colonies at each sampling event. Based on empirical data, literature values, and practical sample handling considerations, we assayed five bees per colony per sampling event. The following equation from Pirk et al. (2013), *N* = ln(1 − *D*) / ln(1 − *P*) (*N* = sample size, ln = natural logarithm, *D* = probability of detection, *P* = proportion of infected bees), predicts that with a sample size of five bees, pathogenic infections affecting 45 % or more of the individuals within a colony would be detected with 95 % probability (Pirk et al. 2013); this sample size has been proven sufficient for the pathogen-specific PCR detection of highly prevalent pathogens (Daughenbaugh et al. 2015; Runckel et al. 2011).

### RNA isolation

Bee samples were homogenized in water using beads (3 mm) and a TissueLyzer (Qiagen) at 30 Hz for 2 min. Samples were centrifuged for 12 min at 12,000*×g* at 4 °C to pellet debris, and RNA from supernatants was extracted using TRIzol reagent (Life Technologies) according to the manufacturer’s instructions (Runckel et al. 2011).

### Reverse transcription/cDNA synthesis

cDNA synthesis reactions were performed by incubating 1,000–2,000 ng total RNA, Moloney murine leukemia virus (M-MLV) reverse transcriptase (Promega), and 500 ng random hexamer primers (IDT) for 1 h at 37 °C, according to the manufacturer’s instructions (Runckel et al. 2011).

### Polymerase chain reaction (PCR)

PCR was performed according to standard methods using the primers listed in Supplemental Table S1 (Runckel et al. 2011). In brief, 1 μL cDNA template was combined with 10 pmol of each forward and reverse primer and amplified with ChoiceTaq polymerase (Denville) according to the manufacturer’s instructions using the following cycling conditions: 95 °C for 5 min; 35 cycles of 95 °C for 30 s, 57 °C for 30 s, and 72 °C for 30 s, followed by final elongation at 72 °C for 4 min. The PCR products were visualized by gel electrophoresis/fluorescence imaging.

### Quantitative PCR (qPCR)

Quantitative PCR was used to analyze the relative abundance of the most prevalent pathogens, which were all RNA viruses, in select samples to investigate the relationship between virus abundance and honey bee colony health. Five hundred nanograms of RNA from each of these samples was reverse transcribed with M-MLV as described above. All qPCR reactions were performed in triplicate with a CFX Connect Real Time instrument (BioRad); reaction conditions and equations for determining the relative abundance based on standard curves are provided in supplemental methods (Online Resource 1).

#### Statistical analysis of PCR

For this study, we use “pathogen prevalence” to refer to the total number of pathogens detected by PCR out of a target list of 16. Though our interest was in the relationship between strength rating and pathogen prevalence, graphical analyses indicated that there were likely relationships between pathogen prevalence and sampling time as well as between strength and sampling time. Thus, we used a Poisson log-linear regression model and accounted for an interaction between sample date (time period), beekeeping operation, colony strength, and pathogen prevalence. Observations with average strength rating were not included in some analyses to simplify the inferences between strong (S) and weak (W). The natural logarithm (ln) of the pathogen prevalence data was used in comparisons between each beekeeping operation and time period combination (Pirk et al. 2013). For the model, we used beekeeping operation 1, before almond pollination (time period 1), and weak colonies as the base level.

In all, our model can be expressed

$$ \begin{array}{c}\hfill {y}_i\sim \mathrm{Poisson}\left({\mu}_i\right)\hfill \\ {}\hfill \log \left({\mu}_i\right)={\beta}_0+{\beta}_1\times \mathrm{operation}\ {2}_i+\hfill \\ {}\hfill {\beta}_2\times \mathrm{operation}\ {3}_i+{\beta}_3\times {\left(S:\mathrm{period}\ 1\right)}_i+\hfill \\ {}\hfill {\beta}_4\times {\left(\mathrm{W}:\mathrm{period}\ 2\right)}_i+{\beta}_5\times {\left(\mathrm{S}:\mathrm{period}\ 2\right)}_i+\hfill \\ {}\hfill {\beta}_6\times {\left(\mathrm{W}:\mathrm{period}\ 3\right)}_i+{\beta}_7\times {\left(\mathrm{S}:\mathrm{period}\ 3\right)}_i+\hfill \\ {}\hfill {\beta}_8\times {\left(\mathrm{W}:\mathrm{period}\ 4\right)}_i+{\beta}_9\times {\left(\mathrm{S}:\mathrm{period}\ 4\right)}_i\hfill \end{array} $$

- where *y*_*i*_ = the total abundance/prevalence for the *i*th observation *i* = 1, 2, …, 180.
- Operation2_*i*_ = 1 if observation *i* came from beekeeping operation 2 and 0 otherwise.
- Operation3_*i*_ = 1 if observation *i* came from beekeeping operation 3 and 0 otherwise.
- Period 2_*i*_ = 1 if observation *i* was taken during and 0 otherwise.
- Period 3_*i*_ = 1 if observation *i* was taken after pollination and 0 otherwise.
- Period 4_*i*_ = 1 if observation *i* was taken in the second after pollination sampling time and 0 otherwise.
- *A*_*i*_ = 1 if observation *i* was average (colony strength) and 0 otherwise.
- *S*_*i*_ = 1 if observation *i* was strong (colony strength) and 0 otherwise.

#### Statistical analysis of qPCR

The relationship between colony strength rating and pathogen abundance was evaluated using a log-normal regression to model the total abundance with the predictor of interest, strength rating, while also accounting for the different beekeeping operations and the sampling time period. To evaluate if the relative abundance of the most prevalent pathogens, which were all (+) sense RNA viruses (i.e., BQCV, SBV, LSV1, and LSV2), we utilized virus copy number as an indication of infection severity, though the relationship between virus copy number and virus associated disease or affects on the honey bee host are largely unknown (de Miranda et al. 2013). Pathogen abundance was defined as the summed abundance of the RNA virus genome copy numbers, which were measured by qPCR. Samples that did not test positive for the virus by PCR were not assessed by qPCR and given a value of zero. In total, there were 53 observations of total abundance after inputting zeros for negative PCR tests. A log-linear regression was used to model the total abundance with the predictor of interest, strength rating, while also accounting for the different beekeeping operations and the sampling time period; 1 was added to each observation since some observations had 0 total abundance. Some bee colonies were measured multiple times in the 53 observations. We accounted for these repeated measures on colonies with a random effect for colony, but found the variance between colonies to be minimal compared to the overall variance.

$$ \begin{array}{c}\hfill \log \left({y}_i\right)={\beta}_0+{\beta}_1\times \mathrm{operation}\ {2}_i+\hfill \\ {}\hfill {\beta}_2\times \mathrm{operation}\ {3}_i+{\beta}_3\times \mathrm{period}\ {2}_i+\hfill \\ {}\hfill {\beta}_4\times \mathrm{period}\ {3}_i+{\beta}_5\times \mathrm{period}\ {4}_i+\hfill \\ {}\hfill {\beta}_6\times {S}_i+{\beta}_7\times {A}_i+{\gamma}_{j(i)}+{\epsilon}_i\hfill \end{array} $$

here *γ*_*j*(*i*)_ is the random effect for colony. We assume *γ*_*j*(*i*)_ ∼ *N*(0, *σ* ^2^_colony_), *ϵ*_*i*_ ∼ *N*(0, *σ* ^*2*^_*y*_), and *γ*_*j*(*i*)_ and *ϵ*_*i*_ are independent for all *j* = 1, 2, …, 60, *i* = 1, 2, 3, …, 180. Variables are defined as they were in Section 2.6.1. Coefficients of interest for this study are *β*_0_, *β*_6_, and *β*_7_. Estimated means and standard errors for these coefficients of interest were obtained using the regression output. Models were fit using the software R (Core Team 2014), the package lme4 was used for the mixed model described in Section 2.6.1 (Bates et al. 2015).

## Results

### Honey bee colony monitoring and pathogen diagnostics

Commercially managed colonies from three Montana-based beekeeping operations were monitored before, during, and after the almond pollination season (i.e., October 2013 to June 2014). Colony population size was utilized as a proxy for colony health and monitored at each sampling event. At the onset of the study, each beekeeping operation selected weak, average, and strong honey bee colonies that were monitored throughout the study. These colonies were located in Montana before and after almond pollination and in California during the almond pollination season (February 2014) (Figure 1). Honey bee colony strength, pathogen prevalence, and abundance were monitored throughout the study.

#### Pathogen detection

To identify the pathogens associated with the honey bee samples collected over the course of this study, we utilized PCR to test for a suite of 16 common pathogens. The majority of these pathogens were viruses including Lake Sinai virus 1 (LSV1), LSV2, LSV3, LSV4, LSV5, Black queen cell virus (BQCV), DWV, SBV, ABPV, CBPV, IAPV, and KBV. In addition, we tested for bacterial pathogens (i.e., *P. larvae* and *M. plutonius*) and eukaryotic parasites including the microsporidia *Nosema* spp. and trypanosomatids (i.e., *C. mellificae SF */* L. passim*). In order to identify the most prevalent pathogens associated with each sample (i.e., affecting 45 % or more of the individuals within a colony), five bees per sample were utilized for pathogen analyses (Daughenbaugh et al. 2015; Pirk et al. 2013). Pathogen-specific PCR was performed to identify the pathogens associated with each sample (Supplemental Tables S1 and S2 and Supplemental Figures S1 and S2). In this work, we use “pathogen occurrence” to indicate the frequency of detecting a specific pathogen as a percentage of the total number of positive tests, “pathogen prevalence” to indicate the number of different pathogens detected in a sample, and “total pathogen abundance” to describe the total number of pathogen genome copies in select samples.

**Figure 2.**
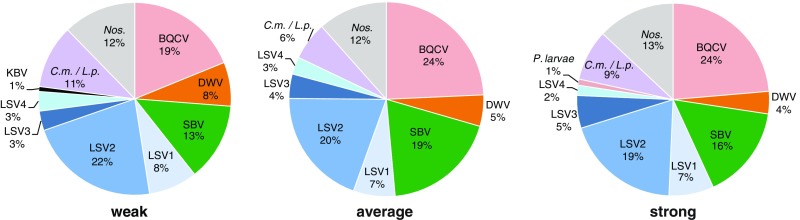
Distribution of honey bee pathogens detected in weak, average, and strong colonies. Honey bee samples were obtained from monitor colonies from October 2013 to June 2014. PCR was used to test for 16 honey bee-infecting pathogens including viruses (ABPV, BQCV, CBPV, DWV, IAPV, KBV, SBV, LSV1, LSV2, LSV3, LSV4, and LSV5, microsporidia (*N. ceranae*), bacteria (*P. larvae* and *M. plutonius*), and trypanosomatids (*C.m*./*L.p*.). The pathogen occurrence in weak (<5 frames; *n* = 41), average (six to eight frames), or strong (>9 frames; *n* = 81) honey bee colonies is shown as a percentage of the total number of pathogens detected by PCR. The percent pathogen occurrence for each colony strength rating is as follows: weak (BQCV 19 %, DWV 8 %, SBV 13 %, LSV1 8 %, LSV2 22 %, LSV3 3 %, LSV4 3 %, KBV 1 %, *C.m./L.p.* 11 %, *N. ceranae* 12 %), average (BQCV 24 %, DWV 5 %, SBV 19 %, LSV1 7 %, LSV2 20 %, LSV3 4 %, LSV4 3 %, *C.m./L.p.* 6 %, *N. ceranae* 12 %), and strong (BQCV 24 %, DWV 4 %, SBV 16 %, LSV1 7 %, LSV2 19 %, LSV3 5 %, LSV4 2 %, *P. larvae* 1 %, *C.m./L.p.* 9 %, *N. ceranae* 13 %).

To identify the most common pathogens detected in this study, we calculated the occurrence of each pathogen as a percentage of the total number of positive tests for each colony strength category (Figure 2). Throughout this study, 176 samples with corresponding colony strength observations were obtained (Supplemental Table S2). Colony strength observations were unevenly distributed between weak, average, and strong colony ratings (i.e., weak colony strength was observed at 41 sampling events, average colony strength was observed at 54 sampling events, and strong colony strength was observed at 81 sampling events). In addition, the total number of pathogen-specific PCR tests varied with each category. Specifically, weak colonies had 122 positive tests, average colonies had 178 positive tests, and strong colonies had 292 positive tests. Therefore, on average across all sampling dates, weak colonies had 2.98 pathogens per sample, average strength colonies had 3.30 pathogens per sample, and strong colonies had 3.60 pathogens per sample. The occurrence (frequency of detection) varied by pathogen (Figure 2). The most readily detected pathogens in this sample cohort were the Lake Sinai viruses (LSV1, LSV2, LSV3, and LSV4), which accounted for 36 % of the total positive tests in weak colonies, 34 % in average colonies, and 33 % in strong colonies (Figure 2). Overall, the most frequently detected pathogens were BQCV, LSV2, SBV, *Nosema ceranae*, *C. mellificae / L. passim*, and LSV1 (Figure 2).

**Figure 3.**
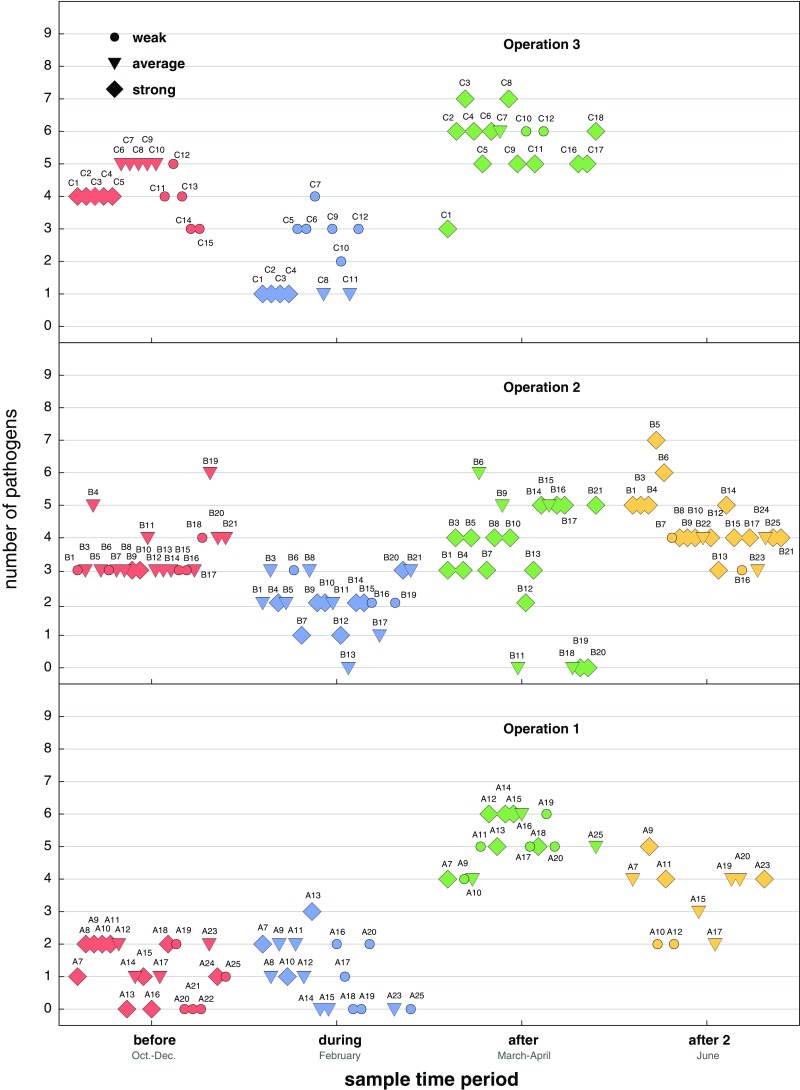
Colony health and pathogen prevalence before, during, and after almond pollination for each beekeeping operation. Honey bee samples were obtained from monitor colonies from October 2013 to June 2014. PCR was used to test for 16 honey bee-infecting pathogens. Pathogen prevalence (*y*-axis) refers to the number of different pathogens detected in a sample. Colony strength was monitored as a proxy for colony health; weak (<5 frames), average colonies (6 - 8 frames), or strong (>9 frames). Each colony was assigned a unique identifier (e.g., “C1” for colony number 1 in beekeeping operation 3); icon size and shape were used to graphically illustrate colony strength over the course of the study (*x*-axis).

Graphical analyses of pathogen prevalence vs. colony strength rating and time revealed that each beekeeping operation had different pathogen prevalence levels and that the relationship between colony strength and pathogen prevalence varied at different sampling times (Figure 3). For example, the mean pathogen prevalence was in general greater in samples obtained immediately after almond pollination (after) for all beekeeping operations, but the differences were more striking for beekeeping operations 1 and 3 (Figure 3). In general, pathogen prevalence was largest in the samples obtained after almond pollination and lowest in samples obtained during almond pollination for all operations (Figure 3, Supplemental Table S2, and Supplemental Figures S1 and S2).

To examine the relationship between colony strength and pathogen prevalence, a Poisson log-linear regression was used to compare the mean number of pathogens present in strong vs. weak colonies (Table I). This model accounted for potential differences in each beekeeping operation, as well as the potential interaction between time period and colony strength. The analyses presented herein are focused on comparisons between weak and strong colonies. The natural logarithm (ln) of the pathogen prevalence data was used in comparison between the mean pathogen prevalence in weak and strong colonies for each beekeeping operation at each sampling event (Pirk et al. 2013). A statistical model was used to calculate the estimated differences between the pathogen prevalence in weak and strong honey bee colonies and associated confidence intervals, based on the standard errors of the differences, given the covariates (Table I, Section 2). For the majority of the sampling dates, the relationship between pathogen prevalence and colony strength indicated that weak colonies had slightly greater pathogen prevalence than strong colonies (i.e., multiplicative differences >1), whereas this trend was reversed in the last sampling period, (i.e., after two had a multiplicative difference <1) (Table I). However, these observed trends could not be differentiated from the expected variation. The largest, though non-significant, difference between the mean number of pathogens in weak colonies, as compared to strong colonies, after accounting for beekeeper, occurred immediately after almond pollination (Table I). Samples obtained from weak colonies directly after almond pollination (March–April) had an estimated 31 % larger mean pathogen number than strong colonies, with an associated 95 % confidence interval of 11 % less to 93 % more after accounting for beekeeping operation (Table I). In addition to mean pathogen prevalence, we investigated if particular pathogens were present at a higher proportion in colonies of different strengths (i.e., weak, strong, and average) (Supplemental Figure S2). This summary is a useful overall representation of the data, but since the results for each pathogen seem to depend solely upon the sampling date, we could not make statistical claims regarding the apparent association of any pathogen with colony strength.

**Table I:** Estimated multiplicative difference in pathogen prevalence from strong to weak colonies in designated sampling period.

Time period	Estimated multiplicative difference	Lower 95 %	Upper 95 %
Before (October–December)	1.05	0.66	1.66
During (February)	1.17	0.68	2.03
After (March–April)	1.31	0.89	1.93
After 2 (June)	0.65	0.34	1.23

**Figure 4.**
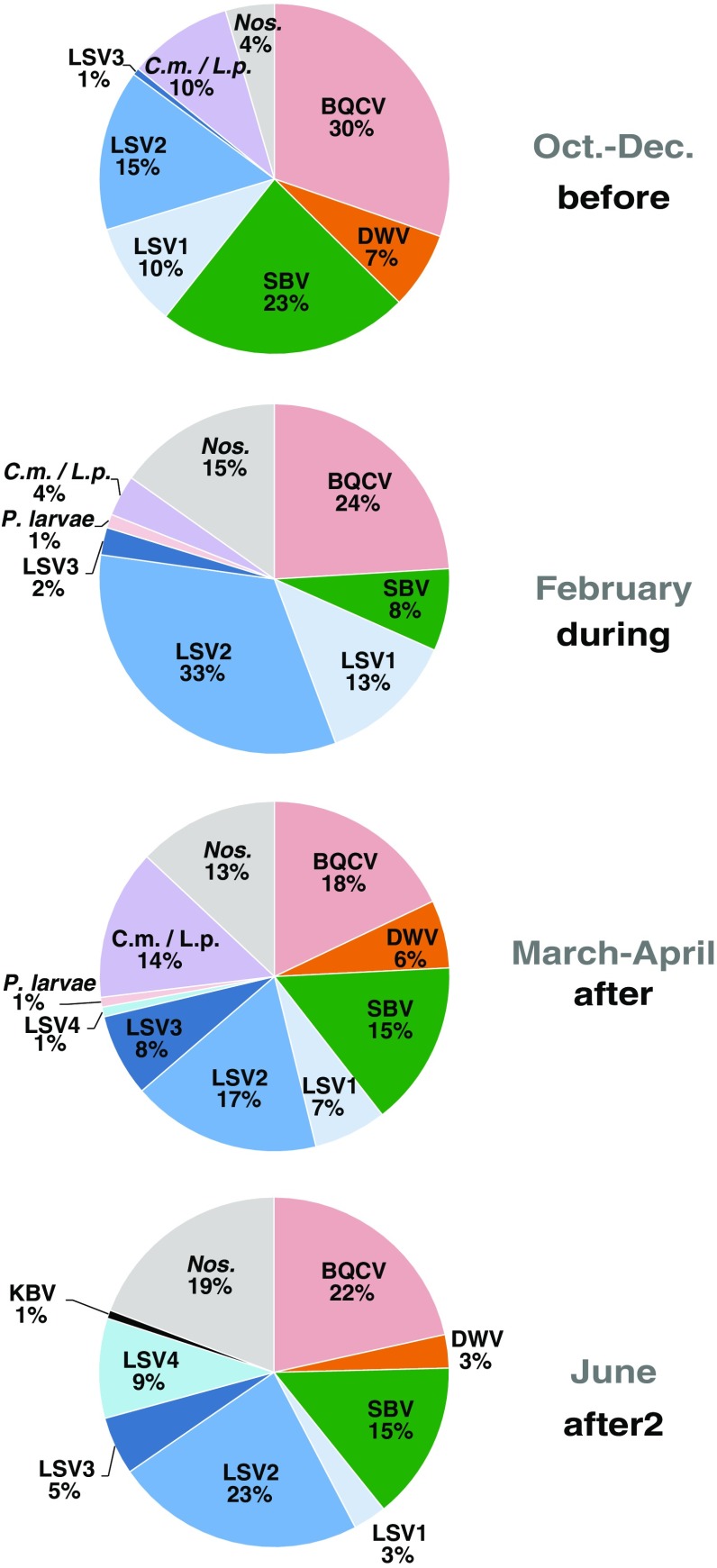
Distribution of honey bee pathogens detected in colonies before, during, and after almond pollination. Honey bee samples were obtained from monitor colonies from October 2013 to June 2014. PCR was used to test for 16 honey bee-infecting pathogens including: viruses (ABPV, BQCV, CBPV, DWV, IAPV, KBV, SBV, LSV1, LSV2, LSV3, LSV4, and LSV5, microsporidia (*N. ceranae)*, bacteria (*P. larvae* and *M. plutonius*), and trypanosomatids (*C.m.*/*L.p.*). Pathogen occurrence, the frequency of detecting a specific pathogen as a percentage of the total number of positive tests, was assessed at different time periods throughout the study. There were 155 positive tests in bee samples collected before almond pollination (October–December 2013), 79 positive tests during almond pollination (February), 223 positive tests immediately after almond pollination (March–May), and 130 positive tests from 20 colonies that were sampled in June 2014.

To determine which pathogens were most common at each time point, we calculated the percent occurrence of each pathogen (Figure 4). The total number of pathogen-specific PCR positive tests varied for each time period. Specifically, there were 155 positive tests in bee samples collected before almond pollination, 79 positive tests during almond pollination, 223 positive tests immediately after almond pollination, and 130 positive tests from 20 colonies that were sampled in June. This representation of the data indicates that BQCV, LSV2, and SBV were more common than other honey bee pathogens including DWV, *N. ceranae*, LSV3, *C. mellificae*/*L. passim*, *P. larvae*, and KBV. The relative percentages of each pathogen vary with the time of sampling (e.g., LSV2 reached peak detection levels during almond pollination); however, pathogen occurrence also varied for each beekeeping operation (Supplemental Figure S1).

#### Pathogen abundance

**Figure 5.**
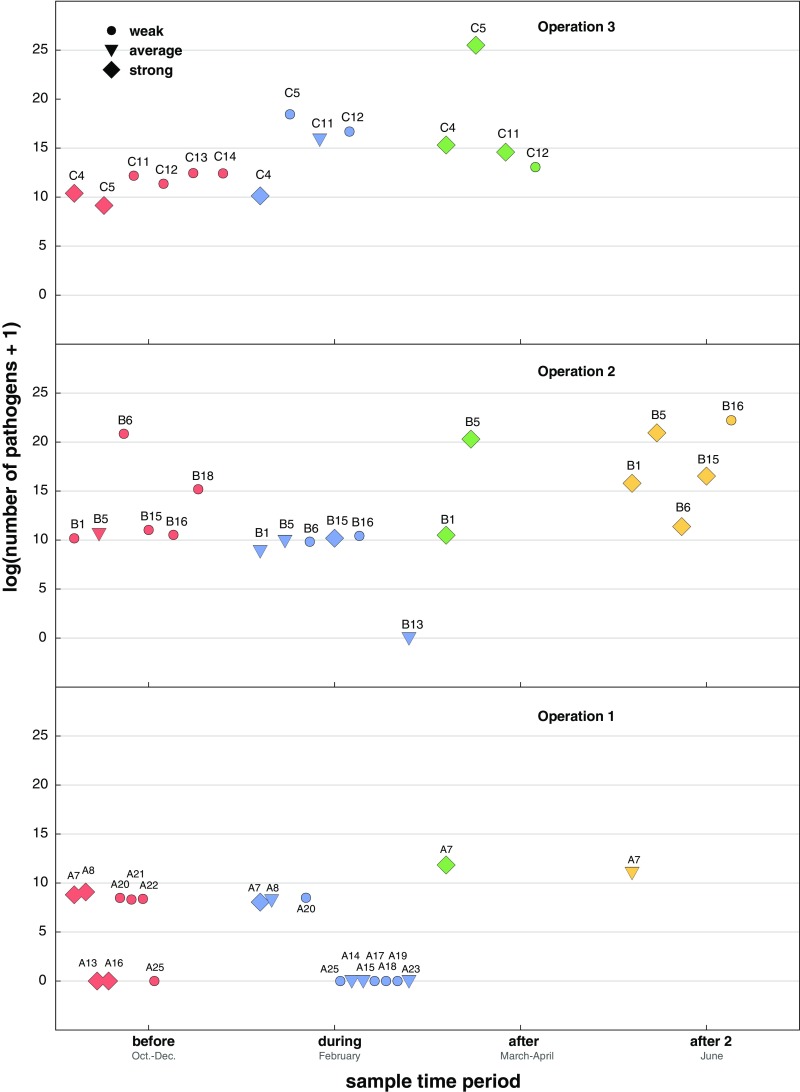
Colony health and pathogen abundance before, during, and after almond pollination for each beekeeping operation. Honey bee samples were obtained from monitor colonies from October 2013 to June 2014. Quantitative PCR (qPCR) was used to determine the abundance of BQCV, LSV2, LSV1, and SBV; total pathogen abundance is the sum of these values (*y*-axis = log natural of total pathogen abundance). In total, there were 53 observations of total abundance in a subset of samples analyzed by qPCR. Each sample is represented by its unique colony identifier (e.g., “C4” for colony number 4 in beekeeping operation 3); icon size and shape were used to graphically illustrate colony strength over the course of the study, October 2013–June 2014 (*x*-axis).

To examine if total pathogen abundance was associated with colony strength, we performed qPCR for the most commonly detected pathogens in our sample cohort, which were all RNA viruses (i.e., BQCV, LSV2, LSV1, and SBV) (Supplemental Table S2). In total, there were 53 observations of total abundance with values that ranged from 0 to 1.19 × 10^11^; total pathogen abundance values were log natural (ln) transformed after adding 1 (Figure 5). We used log-linear regression to model the total abundance with the predictor of interest, strength rating, while also accounting for the different beekeeping operations and the sampling time period and determined that the variance among colonies was minimal compared to the overall variance (estimates and their associated confidence intervals provided (Table II)).

**Table II:** Estimated multiplicative differences in the pathogen abundance between beekeeping operations, at different sampling time periods, and between colonies of different strengths; natural log scale.

	Estimate	Lower bound	Upper bound
Intercept	5.08	2.32	7.84
Operation 2 vs. operation 1	6.90	3.54	10.27
Operation 3 vs. operation 1	8.73	5.13	12.34
During (February) vs. before (October–December)	−0.79	−3.50	1.91
After (March–April) vs. before (October–December)	4.71	0.92	8.50
After2 (June) vs. before (October–December)	6.42	2.44	10.41
Average vs. weak	−2.72	−6.25	0.82
Strong vs. weak	−1.71	−4.77	1.35

#### Colony mortality

The overall colony mortality in this study was 14.8 %. Colony loss varied with the time of sampling and beekeeping operation (Table III). Colony mortality was greatest during almond pollination, during which 13 % of the colonies died. Colony mortality varied by beekeeping operation; specifically, operation 2 experienced only 5.0 % loss, operation 1 had 15.8 % loss, and operation 3 experienced 20 % loss during almond pollination. Colony losses in this sample cohort were less than average US colony losses, which have been ~33 % since 2006 (Steinhauer et al. 2015).

**Table III:** Percentage of honey bee colony mortality by beekeeping operation and time period.

	Time period/date				
Beekeeping operation	Before (October–December)	During (February)	After (March–April)	After2 (June)	Overall
Operation 3	0 %	20.0 %	0 %	0 %	20.0 %
Operation 2	0 %	5.0 %	0 %	0 %	5.0 %
Operation 1	0 %	15.8 %	6.7 %	0 %	21.1 %
Total	0 %	13.0 %	2.0 %	0 %	14.8 %

## Discussion

Honey bees play an important role as pollinators of numerous agricultural crops. The majority of commercially managed honey bee colonies in the US are transported throughout the year to meet the pollination demands of crops including California almond production. Commercial beekeepers in the US and some parts of Europe have experienced high annual average losses, though the factors most responsible for these losses are not well understood. The objectives of this study were to identify the pathogens currently circulating in Western US-based commercial honey bee colonies involved in almond pollination and to examine the relationship between colony strength, pathogen prevalence, and abundance. Pathogen incidence and abundance have been associated with CCD-affected colonies and colony losses in the US (Chen et al. 2014; Cornman et al. 2012; Cox-Foster et al. 2007; Daughenbaugh et al. 2015; Li et al. 2013; van Engelsdorp et al. 2009), Canada (van der Zee et al. 2012), Austria (Berényi et al. 2006), Denmark (Nielson et al. 2008), Luxembourg (Clermont et al. 2014) and Belgium (Ravoet et al. 2013). Therefore, we hypothesized that weak colonies would harbor a greater number of pathogens, as well as have greater loads of these pathogens. Surprisingly, there have been few honey bee colony monitoring studies in the US; therefore, the data presented herein provide an important examination of colony health and pathogen prevalence and abundance in Western US-based honey bee colonies involved in almond pollination.

There are multiple biotic and abiotic factors that contribute to honey bee colony health and survival. The focus for this study was pathogen prevalence and abundance, since several studies indicate that pathogens affect colony health. Specifically, one US-based study that compared multiple variables in CCD and non-CCD-affected colonies determined that weak or dead colonies were more likely to neighbor other weak or dead colonies and that CCD-affected samples had a higher pathogen incidence than controls, whereas pesticide levels were comparable (van Engelsdorp et al. 2009). Similarly, metagenomic analysis of those samples suggested that IAPV abundance was related to colony health (Cox-Foster et al. 2007), though later studies indicated that pathogen number, DWV, and a combination of other pathogens were more positively correlated with CCD-affected colonies (i.e., ABPV, *N* *osema apis*, KBV, LSV1, and LSV2) (Chen and Evans 2007; Johnson et al. 2009; Cornman et al. 2012). More recently, a study that involved ten colonies determined that IAPV was more abundant in colonies with less food stores and less brood/developing bees (Chen et al. 2014). In addition, data from a small-scale monitoring project that also involved only ten colonies suggested that LSV2, LSV1, BQCV, and *N. ceranae* were more abundant in weak colonies, as compared to strong colonies (Daughenbaugh et al. 2015). Though the exact cause(s) of CCD and colony loss remain unknown, these US-based studies suggest that pathogens are an important factor.

Longitudinal monitoring studies are required to understand the key factors affecting honey bee colony losses. These studies help to establish a baseline for typical pathogen incidence and abundance in honey bee colonies throughout the year. The most prevalent pathogens detected in this study were BQCV, LSV2, SBV, *N. ceranae*, and *C. mellificae / L. passim.* Lake Sinai viruses (i.e., LSV2, LSV1, LSV3, and LSV4) accounted for a large proportion of the positive tests. One of the first US-based longitudinal monitoring studies involved 20 colonies managed by a single large-scale commercial beekeeping operation (Runckel et al. 2011). This study indicated that pathogen status within healthy colonies is dynamic and seasonal, and it and other studies have indicated that several samples, obtained at specified time intervals, provide a more accurate depiction of colony health than a single time point. Longitudinal sampling and analysis revealed increased incidence of particular viruses in most colonies within short time periods, resulted in the discovery of the Lake Sinai viruses, and facilitated the isolation and sequencing of a honey bee-infecting trypanosomatid (originally designated *C. mellificae*, *strain SF* but recently re-classified and re-named *L. passim* (Runckel et al. 2011, 2014; Schwarz et al. 2015). However, monitored honey bee colonies remained healthy throughout the study, so it did not provide the sample cohort required to associate pathogens with health status and disease. Similarities between the results of the 2009–2010 sample cohort (Runckel et al. 2011) and the 2013–2014 sample cohort described herein include high prevalence of LSVs, particularly LSV2 during almond pollination, low occurrence of DWV, IAPV, and KBV, and frequent detection of BQCV and SBV. In general, pathogen prevalence was largest in the samples obtained after almond pollination and lowest in samples obtained during almond pollination for all operations; this observation may stem from the seasonality of pathogenic infections (Chen and Siede 2007; Evans and Schwarz 2011; Runckel et al. 2011).

US-wide surveys, including those performed by the Bee Informed Partnership, provide an important overview of the status of colonies and pathogens (reviewed in Steinhauer et al. 2015). However, these surveys rely on voluntary sample submission over the course of the year and are not sufficiently controlled to investigate subtle relationships between colony health and particular biotic or abiotic factors. Monitoring projects outside of the US are informative and serve as models for future studies. The German Bee Monitoring project is an excellent example of a longitudinal monitoring study that assessed the role of management practices, pathogens, pesticides, and environmental factors, in periodic high winter losses of honey bee colonies over a period of 4 years (Genersch et al. 2010; Gisder et al. 2010). That study identified several factors that were significantly related to honey bee colony loss including high levels of *Varroa destructor* mites, queen age, DWV and ABPV infection, and weak colony strength rating in autumn (Genersch et al. 2010). Likewise, the prevalence and seasonal variation of virus infections and mite infestation was assessed in a 1-year country-wide study in France that involved 360 honey bee colonies from 36 apiaries sampled in the spring, summer, and autumn (Tentcheva et al. 2004). PCR was used to assess the incidence of six bee viruses; DWV was the most prevalent followed by BQCV and SBV, and the detection frequency varied over the course of the year (Tentcheva et al. 2004). Recently, the distributions of ABPV, BQCV, CBPV, DWV, SBV across all districts of Croatia were examined to provide an epidemiological baseline (Gajger et al. 2014). DWV was the most readily detected virus, whereas KBV and IAPV were not detected (Gajger et al. 2014). Several studies indicate that the combination of DWV infection and *V. destructor* mite infestation negatively affect colony health (reviewed in Nazzi et al. 2012). DWV prevalence was very low in our study, thus its relationship with colony health could not be assessed.

The results from this cohort study indicate that the relationship between pathogen incidence and abundance is complex and dependent upon beekeeping operation and sampling time. The data presented herein are similar to other studies in that they do not rule out a relationship between colony strength and pathogen prevalence. Large variability in this data set suggests that future studies should include larger sample sizes, involve more beekeeping operations, and incorporate standardized management practices. Colony mortality in this study was lower than the average losses calculated from recent US national survey data (14.8 % vs. 33 % respectively), though the reason(s) for low colony mortality remain unknown. Future studies that closely monitor additional factors, such as foraging success and diversity via pollen traps, agrochemical exposure via chemical analysis, and mite abundance, in conjunction with colony health and pathogen prevalence and abundance will help elucidate the factors that most correlate with colony health and survival.

## Electronic supplementary material

Below is the link to the electronic supplementary material.

ESM 1 (DOCX 40 kb)

ESM 2 (PDF 598 kb)

ESM 3 (PDF 159 kb)

ESM 4 (PDF 123 kb)

ESM 5 (XLSX 65 kb)

ESM 6 (PDF 1.64 mb)
