# Supplementary material for: Pathogen prevalence and abundance in honey bee colonies involved in almond pollination
Source: Apidologie. 2015 Oct 21;47(2):251–66. doi: 10.1007/s13592-015-0395-5 (PMC4766222; doi:10.1007/s13592-015-0395-5)
Supplement: Supplementary file 1 — (DOCX 40 kb) [file 13592_2015_395_MOESM1_ESM.docx]

Apidologie Online Ressource 1

**Pathogen prevalence and abundance in honey bee colonies involved in almond pollination**

Ian Cavigli^1^, Katie Daughenbaugh^1^, Madison Martin^1^, Michael Lerch^4^, Katie Banner^4^, Emma Garcia^1^, Laura M. Brutscher^1,2,3^, and Michelle L. Flenniken*^,1,2^

^1^Department of Plant Sciences and Plant Pathology, ^2^Institute on Ecosystems, ^3^Department of Microbiology and Immunology, ^4^Department of Mathematical Sciences, Montana State University, Bozeman, MT, USA, 59717

*corresponding author: Michelle L. Flenniken, Department of Plant Sciences and Plant Pathology, Montana State University, Bozeman, MT 59717 U.S.A.

**Supporting Methods**

**2.1. Longitudinal monitoring/sampling of commercially managed honey bee colonies**

Three Montana-based (Broadwater, Yellowstone, Treasure counties) commercial beekeeping operations that transport their honey bee colonies ~ 1,200 miles to California (Merced and Stanislaus counties) each winter for the almond bloom provided honey bee samples before, during, and after almond pollination (February 2014) (Figure 1). Colony health, using colony population size as a proxy, was assessed by the number of frames covered with honey bees (frame counts) at each sampling event (Delaplane and van der Steen 2013; OSU 2011). Colony strength was defined as follows: weak colonies (< 5 frames covered with bees), average colonies (6-8 frames covered with bees), and strong colonies (> 9 frames covered with bees). Live honey bee samples (~ 100 per sample) were obtained from the top of the frames in the middle of the colony. Samples were composed of female bees of mixed age, including nurse, worker, and forager bees. The samples were collected on ice or dry ice, stored at -20°C, shipped on dry ice, and transferred to -80°C prior to analysis. At the onset of the study in November 2013, each beekeeper identified 15 – 20 colonies of differential health. Specifically, Operation 3 initiated the study with 5 weak, 5 average, and 5 strong colonies and provided samples at three time points; Operation 2 initiated the study with 5 weak, 13 average, and 2 strong colonies and provide samples at 4 time points; and Operation 1 initiated the study with 5 weak, 4 average, and 10 strong colonies and provide samples at 4 time points (Supplemental Table S2). A total of 176 honey bee samples with corresponding colony strength observations were obtained and analyzed, 4 observations of colony strength lacked corresponding samples, and 8 of the original colonies died during the course of this study (Supplemental Table S2). Operation 3 manages ~7,100 colonies, Operation 2 manages ~12,500 colonies, and Operation 1 manages ~3,500 colonies. All the colonies in this study were fed protein patties either before (Operation 3 – November and January, Operation 2 – November) or during almond pollination (Operation 1). In addition, Operation 2 treated monitor colonies with an antifungal treatment (Fumagilin-B®) and fed them Pro-Health®. After almond pollination in May 2014, Operation 2 treated for *Varroa destructor* mites (i.e., two strips of Apivar® per colony), fed pollen patties, and treated some colonies with an antibiotic (Terramycin®). Operation 1 treated some colonies with Bee Shield® before almond pollination (December 2013), and fed colonies essential oils in addition to protein patties immediately before the almond bloom. Operation 1 also treated some colonies with the antibiotic Terramycin® immediately after almond pollination, and fed sugar syrup and essential oils and treated for mites prior to the last sample date in May 2014.

**2.2. Honey bee samples**

Five female bees from each sample were used for RNA extraction, cDNA synthesis, pathogen-specific PCR, and qPCR (Runckel, Flenniken et al. 2011). There are varying recommendations of the number of honey bees required to adequately assess the pathogens associated with a single honey bee colony at a particular point in time (Chen et al. 2014; de Miranda et al. 2013; Genersch et al. 2010; Pirk et al. 2013). Successful pathogen detection is dependent upon sensitivity of the assay and signal to noise ratio of each sample (i.e., pathogen RNA to bee RNA ratio). The objective for pathogen screening in our study was to identify the most prevalent pathogens associated with honey bees sampled from individual colonies at each sampling event. Based on empirical data, literature values, and practical sample handling considerations, we assayed five bees per colony per sampling event. The following equation from Pirk et al. 2013, N = ln(1-D) / ln(1-P) (N=sample size, ln=natural logarithm, D=probability of detection, P=proportion of infected bees) predicts that with a sample size of five bees, pathogenic infections affecting 45% or more of the individuals within a colony would be detected with 95% probability (Pirk et al. 2013); this sample size has proven sufficient for the pathogen-specific PCR detection of highly prevalent pathogens (Daughenbaugh et al. 2015; Runckel, Flenniken et al. 2011).

**2.3. RNA isolation**

Bee samples were homogenized in 800 μL sterile H_2_O with sterile beads (3 mm) using a TissueLyzer (Qiagen) at 30 Hz for 2 min. Bee samples were centrifuged for 12 min at 12,000*xg* at 4°C to pellet debris, and supernatants were transferred to new 1.5 mL tubes containing an equal volume of Trizol reagent (Life Technologies). RNA was extracted according to the manufacturer’s instructions and was suspended in sterile water.

**2.5. Polymerase Chain Reaction (PCR)**

PCR was performed according to standard methods (Runckel, Flenniken et al. 2011, de Miranda et al. 2013; Govan et al. 2000; Lanzi et al. 2006; Maori et al. 2007). In brief, 1 μl cDNA template was combined with 10 pmol of each forward and reverse primer, and amplified with ChoiceTaq polymerase (Denville) according to the manufacturer’s instructions using the following cycling conditions: 95°C for 5 min; 35 cycles of 95°C for 30 s, 57°C for 30 s, and 72°C for 30s, followed by final elongation at 72°C for 4 min. The PCR products were visualized by gel electrophoresis/fluorescence imaging. To minimize the number of negative PCR-tests, we pooled samples (<10) for initial PCR analysis and then assessed individual samples for each pathogen detected in pooled analysis. Positive and negative control reactions were included for each set of reactions.

**2.6. Quantitative PCR (qPCR)**

Quantitative PCR was used to analyze the relative abundance of the most prevalent pathogens in select samples to investigate the relationship between pathogen abundance and honey bee colony health. Five hundred ng of RNA from each of these samples was reverse transcribed with M-MLV as described above. All qPCR reactions were performed in triplicate with a CFX Connect Real Time instrument (BioRad) and the following reaction conditions: 2 μL of cDNA template in 20 μL reactions containing 1X ChoiceTaq Mastermix (Denville), 0.4 µM each forward and reverse primer, 1X SYBR Green (Life Technologies), and 3 mM MgCl2. The qPCR thermo-profile consisted of a single pre-incubation 95°C (1 min), 40 cycles of 95°C (10 s), 58°C (20 s), and 72°C (15 s). Plasmid standards, containing from 10^9^ to 10^3^ copies per reaction, were used as qPCR templates to assess primer efficiency and quantify the relative abundance of each pathogen. The linear standard equations generated by plotting the crossing point (Cp) versus the log_10_ of the initial plasmid copy number for each primer set were as follows: LSV2: y = -3.8147x + 44.805, R^2^ = 0.980; BQCV: y = -3.7336x+ 42.849, R^2^ = 0.996; LSV1: y = -3.1994x + 38.71, R^2^ = 0.982, and SBV: y = -3.3768x + 39.484, R^2^ = 0.996. In addition, qPCR of a host encoded gene, *Apis m*. Rpl8, was performed using 2 μL cDNA template on each qPCR plate to ensure consistency and cDNA quality. qPCR products were analyzed by melting point analysis and 2% agarose gel electrophoresis.

**2.7.1. Statistical analysis of PCR**

For this study, we use “pathogen prevalence” to refer to the total number of pathogens detected by PCR out of a target list of 16. Though our interest is in the relationship between strength rating and pathogen prevalence, graphical analyses indicated that there were likely relationships between pathogen prevalence and sampling time as well as between strength and sampling time. Thus, we used a Poisson log-linear regression model and accounted for an interaction between sample date (time period), beekeeping operation, colony strength, and pathogen prevalence. Colonies were evaluated and sampled multiple times (i.e., either 3 or 4 times), but since subsequent measurements on a single colony were both temporally and geographically distant and graphical analyses did not suggest a relationship between pathogen prevalence and subsequent measures on a given colony, this parameter was not included in our model. Observations with average strength rating were not included in the analysis to simplify the inferences between strong (S) and weak (W). The natural logarithm (ln) of the pathogen prevalence data was used in comparisons between each beekeeping operation and time period combination; for the model, we used beekeeping Operation 1, before almond pollination (time period 1), and weak colonies as the base level.

In all, our model can be expressed

$$y_{i}\sim\mathrm{Poisson}(\mu_{i})$$

$$\log(\mu_{i})=\beta_{0}+\beta_{1}\times{Operation2}_{i}+$$

$$\beta_{2}\times{Operation3}_{i}+\beta_{3}\times(S:period1)_{i}+$$

$$\beta_{4}\times(W:period2)_{i}+\beta_{5}\times(S:period2)_{i}+$$

$$\beta_{6}\times(W:period3)_{i}+\beta_{7}\times(S:period3)_{i}+$$

$$\beta_{8}\times(W:period4)_{i}+\beta_{9}\times(S:period4)_{i}$$

- $y_{i}=$ the total abundance/prevalence for the $i^{th}$ observation $i=1,2,...,180$.
- ${Operation2}_{i}=1$ if observation $i$ came from beekeeping Operation 2 and 0 otherwise.
- ${Operation3}_{i}=1$ if observation $i$ came from beekeeping Operation 3 and 0 otherwise.
- ${period2}_{i}=1$ if observation $i$ was taken during and 0 otherwise.
- ${period3}_{i}=1$ if observation $i$ was taken after pollination and 0 otherwise.
- ${period4}_{i}=1$ if observation $i$ was taken in the second after pollination sampling time and 0 otherwise.
- $A_{i}=1$ if observation $i$ was Average (colony strength) and 0 otherwise.
- $S_{i}=1$ if observation $i$ was Strong (colony strength) and 0 otherwise.

In the equation above, $\gamma_{j(i)}$ is the random effect for colony. We assume $\gamma_{j(i)}\sim N(0,\sigma_{colony}^{2})$, $\epsilon_{i}\sim N(0,\sigma_{y}^{2})$ and $\gamma_{j(i)}$ and $\epsilon_{i}$ are independent for all $j=1,2,...,60$, $i=1,2,3,...,180$. $S$ and $W$ are indicators for strong and weak colony ratings. Here, we defined weak colonies from beekeeping Operation 1 during time period1 (before almond pollination) as the base-level for comparisons. $\mu_{i}$ is the expected pathogen prevalence given the covariates. Since we observed an interaction between time period and strength rating, our question of interest must be evaluated in each of the time periods. Thus our final inferences are based on our estimates of $\text{β}_{\text{3}}$, the difference between $\beta_{4}$ and $\beta_{5}$, $\beta_{6}$ and $\beta_{7}$, and $\beta_{8}$ and $\beta_{9}$; the values for parameters in the equation that are not required to address specific questions become 0.

**References associated with longitudinal monitoring of honey bee colonies.**

Longitudinal monitoring of colony health and pathogen prevalence and abundance is critical to determining the role of pathogens in colony losses (Berényi et al. 2006; Chen et al. 2014; Clermont et al. 2014; de Miranda et al. 2013; Delaplane and van der Steen 2013; Ellis et al. 2010; Gajger et al. 2014; Genersch et al. 2010; Gisder et al. 2010; McMenamin and Genersch 2015; Nielsen et al. 2008; Ravoet et al. 2013; Runckel, Flenniken et al. 2011; Spleen et al. 2013; Steinhauer et al. 2014; Tentcheva et al. 2004; van der Zee et al. 2012; van Engelsdorp et al. 2008; vanEngelsdorp et al. 2012; vanEngelsdorp et al. 2009; vanEngelsdorp et al. 2013).
