# Supplementary material for: Pathogen prevalence and abundance in honey bee colonies involved in almond pollination
Source: Apidologie. 2015 Oct 21;47(2):251–66. doi: 10.1007/s13592-015-0395-5 (PMC4766222; doi:10.1007/s13592-015-0395-5)
Supplement: Supplementary file 2 — (PDF 598 kb) [file 13592_2015_395_MOESM2_ESM.pdf]

Apidologie Supplementary Material (Fig. S1)

# Pathogen prevalence and abundance in honey bee colonies involved in almond pollination

Ian Cavigli, Katie F. Daughenbaugh, Madison Martin, Michael Lerch, Katie Banner, Emma Garcia, Laura M. Brutscher, Michelle L. Flenniken*

*corresponding author: Michelle L. Flenniken, Department of Plant Sciences and Plant Pathology, Montana State University, Bozeman, MT 59717 USA.

**Supplemental Figure S1.** Percent pathogen occurrence at each sampling time point from honey bee colonies in Operation 1: black = proportion of colonies that tested positive for pathogen at that time point.

| | Before | During | After | After2 |
|---|---|---|---|---|
| **BQCV** | 63.16 | 43.75 | 71 | 90 |
| **DWV** | 10.53 | 0 | 100 | 40 |
| **SBV** | 26.32 | 0 | 50 | 40 |
| **AFB** | 0 | 6 | 0 | 0 |
| ***Cm*** | 11 | 0 | 93 | 0 |
| ***Nc*** | 5 | 25 | 100 | 50 |
| **LSV1** | 5.26 | 31.25 | 0 | 20 |
| **LSV2** | 0 | 0 | 100 | 100 |
| **LSV3** | 0 | 0 | 0 | 40 |
| **LSV4** | 0 | 0 | 0 | 70 |

**Supplemental Figure S1.** Percent pathogen occurrence at each sampling time point from honey bee colonies in Operation 2: black = proportion of colonies that tested positive for pathogen at that time point.

| | Before | During | After | After2 |
|---|---|---|---|---|
| **BQCV** | 100 | 60 | 93.75 | 95 |
| **DWV** | 5 | 0 | 0 | 0 |
| **SBV** | 100 | 0 | 93.75 | 70 |
| **AFB** | 0 | 0 | 12.5 | 0 |
| ***Nc*** | 30 | 40 | 12.5 | 100 |
| ***Cm*** | 0 | 15 | 18.75 | 0 |
| **KBV** | 0 | 0 | 0 | 5 |
| **LSV1** | 5 | 0 | 37.5 | 10 |
| **LSV2** | 100 | 70 | 100 | 100 |
| **LSV3** | 0 | 0 | 43.75 | 15 |
| **LSV4** | 0 | 0 | 0 | 30 |

**Supplemental Figure S1.** Percent pathogen occurrence at each sampling time point from honey bee colonies in Operation 3: black = proportion of colonies that tested positive for pathogen at that time point.

| | Before | During | After |
|---|---|---|---|
| **BQCV** | 100 | 0 | 100 |
| **DWV** | 53 | 0 | 0 |
| **SBV** | 73 | 50 | 80 |
| ***Nc*** | 7 | 0 | 87 |
| ***Cm*** | 87 | 0 | 100 |
| **LSV1** | 87 | 42 | 60 |
| **LSV2** | 20 | 100 | 60 |
| **LSV3** | 0 | 8 | 67 |
| **LSV4** | 0 | 0 | 13 |
