# Supplementary material for: Pathogen prevalence and abundance in honey bee colonies involved in almond pollination
Source: Apidologie. 2015 Oct 21;47(2):251–66. doi: 10.1007/s13592-015-0395-5 (PMC4766222; doi:10.1007/s13592-015-0395-5)
Supplement: Supplementary file 3 — (PDF 159 kb) [file 13592_2015_395_MOESM3_ESM.pdf]

Pathogen prevalence and abundance in honey bee colonies involved in almond pollination

Ian Cavigli, Katie F. Daughenbaugh, Madison Martin, Michael Lerch, Katie Banner, Emma Garcia, Laura M. Brutscher, Michelle L. Flenniken*

*corresponding author: Michelle L. Flenniken, Department of Plant Sciences and Plant Pathology, Montana State University, Bozeman, MT 59717 USA.

Supplemental Figure S2. Individual Pathogen Prevalence

A. Summary of the pathogens detected in weak, strong, and average colonies using pathogen-specific PCR. The percentage of samples that tested positive for each pathogen is listed by colony strength.

	weak (n=41)	average (n=54)	strong (n=81)
BQCV	56%	78%	85%
CBPV	0%	0%	0%
DWV	22%	17%	14%
SBV	39%	61%	57%
P. larvae (AFB)	0%	0%	4%
trypanosomatids (*C.m. / L.p.*)	32%	20%	31%
N. ceranae	37%	37%	47%
LSV1	24%	22%	27%
LSV2	66%	63%	70%
LSV3	7%	9%	19%
LSV4	0%	2%	7%
LSV5	0%	0%	0%
IAPV	0%	0%	0%
KBV	0%	0%	1%
M. plutonius	0%	0%	0%

B. Summary of the pathogens detected in weak, strong, and average colonies using pathogen-specific PCR. The percentage of samples that tested positive for each pathogen at each time period (date) is presented in relation to colony strength. This table provides an overall summary of the data. Statistical evaluation of this data was not performed since the results for each pathogen were dependent on time period and there were a significant number of 0's in the data set.

	**weak % (n)**	**average % (n)**	**strong % (n)**	**all colonies % (n)**
**BQCV**				
**before** (Oct-Dec)	70% (15)	100% (22)	90% (17)	87.0% (54)
**during** (Feb)	10% (15)	50% (17)	50% (15)	40.4% (47)
**after** (March-April)	90% (7)	90% (7)	90% (31)	88.9% (45)
**after2** (June)	100% (4)	80% (8)	100% (18)	93.3% (30)
**CBPV**				
**before** (Oct-Dec)	0% (15)	0% (22)	0% (17)	0% (54)
**during** (Feb)	0% (15)	0% (16)	0% (15)	0% (46)
**after** (March-April)	0% (7)	0% (8)	0% (30)	0% (45)
**after2** (June)	0% (4)	0% (8)	0% (18)	0% (30)
**DWV**				
**before** (Oct-Dec)	30% (15)	20% (22)	10% (17)	20.4% (54)
**during** (Feb)	0% (15)	0% (16)	0% (15)	0% (46)
**after** (March-April)	7% (7)	40% (8)	20% (29)	31.8% (44)
**after2** (June)	0% (4)	10% (8)	20% (18)	13.3% (30)
**SBV**				
**before** (Oct-Dec)	40% (15)	100% (22)	50% (17)	66.7% (54)
**during** (Feb)	40% (15)	0% (17)	0% (15)	12.8% (47)
**after** (March-April)	40% (7)	70% (7)	80% (31)	75.6% (45)
**after2** (June)	30% (4)	90% (8)	60% (18)	63.3% (30)
P. larvae				
**before** (Oct-Dec)	0% (15)	0% (22)	0% (17)	0% (54)
**during** (Feb)	0% (15)	0% (17)	10% (15)	2.1% (47)
**after** (March-April)	0% (7)	0% (7)	10% (31)	4.4% (45)
**after2** (June)	0% (4)	0% (8)	0% (18)	0% (30)
	**weak % (n)**	**average % (n)**	**strong % (n)**	**all colonies % (n)**

C.m. / L.p.				
**before** (Oct-Dec)	30% (15)	20% (22)	30% (17)	27.8% (54)
**during** (Feb)	10% (15)	10% (17)	10% (15)	6.4% (47)
**after** (March-April)	100% (7)	70% (7)	60% (31)	68.9% (45)
**after2** (June)	0% (4)	0% (8)	0% (18)	0% (30)
Nosema				
**before** (Oct-Dec)	10% (15)	30% (22)	0% (17)	13.0% (54)
**during** (Feb)	30% (15)	20% (17)	20% (15)	25.5% (47)
**after** (March-April)	100% (7)	70% (7)	60% (31)	64.4% (45)
**after2** (June)	50% (4)	60% (8)	100% (18)	83.3% (30)
**LSV1**				
**before** (Oct-Dec)	20% (15)	30% (22)	40% (17)	27.8% (54)
**during** (Feb)	50% (15)	10% (17)	10% (15)	21.3% (47)
**after** (March-April)	0% (7)	40% (7)	40% (31)	33.3% (45)
**after2** (June)	0% (4)	10% (8)	20% (18)	13.3% (30)
**LSV2**				
**before** (Oct-Dec)	50% (15)	60% (22)	10% (17)	42.6% (54)
**during** (Feb)	50% (15)	40% (17)	70% (15)	55.3% (47)
**after** (March-April)	100% (7)	90% (7)	80% (31)	86.7% (45)
**after2** (June)	100% (4)	100% (8)	100% (18)	100% (30)
**LSV3**				
**before** (Oct-Dec)	0% (15)	10% (22)	0% (17)	1.9% (54)
**during** (Feb)	10% (15)	10% (17)	0% (15)	4.3% (47)
**after** (March-April)	30% (7)	40% (7)	40% (31)	37.8% (45)
**after2** (June)	0% (4)	0% (8)	20% (18)	10.0% (30)
**LSV4**				
**before** (Oct-Dec)	0% (15)	0% (22)	0% (17)	0% (54)
**during** (Feb)	0% (15)	0% (17)	0% (15)	0% (47)
**after** (March-April)	0% (7)	10% (7)	0% (31)	2.2% (45)
**after2** (June)	0% (4)	0% (8)	30% (18)	20% (30)
	**weak % (n)**	**average % (n)**	**strong % (n)**	**all colonies % (n)**
**LSV5**				

**before** (Oct-Dec)	0% (15)	0% (22)	0% (17)	0% (54)
**during** (Feb)	0% (15)	0% (17)	0% (15)	0% (47)
**after** (March-April)	0% (7)	0% (7)	0% (31)	0% (45)
**after2** (June)	0% (4)	0% (8)	0% (18)	0% (30)
**IAPV**				
**before** (Oct-Dec)	0% (15)	0% (22)	0% (17)	0% (54)
**during** (Feb)	0% (15)	0% (17)	0% (15)	0% (47)
**after** (March-April)	0% (7)	0% (7)	0% (31)	0% (45)
**after2** (June)	0% (4)	0% (8)	0% (18)	0% (30)
**ABPV**				
**before** (Oct-Dec)	0% (15)	0% (22)	0% (17)	0% (54)
**during** (Feb)	0% (15)	0% (17)	0% (15)	0% (47)
**after** (March-April)	0% (7)	0% (7)	0% (31)	0% (45)
**after2** (June)	0% (4)	0% (8)	0% (18)	0% (30)
**KBV**				
**before** (Oct-Dec)	0% (15)	0% (22)	0% (17)	0% (54)
**during** (Feb)	0% (15)	0% (17)	0% (15)	0% (47)
**after** (March-April)	0% (7)	0% (7)	0% (31)	0% (45)
**after2** (June)	0% (4)	0% (8)	10% (18)	3.3% (30)
M. plutonius				
**before** (Oct-Dec)	0% (15)	0% (22)	0% (17)	0% (54)
**during** (Feb)	0% (15)	0% (17)	0% (15)	0% (47)
**after** (March-April)	0% (7)	0% (7)	0% (31)	0% (45)
**after2** (June)	0% (4)	0% (8)	0% (18)	0% (30)
