# Supplementary material for: Pathogen prevalence and abundance in honey bee colonies involved in almond pollination
Source: Apidologie. 2015 Oct 21;47(2):251–66. doi: 10.1007/s13592-015-0395-5 (PMC4766222; doi:10.1007/s13592-015-0395-5)
Supplement: Supplementary file 4 — (PDF 123 kb) [file 13592_2015_395_MOESM4_ESM.pdf]

Pathogen prevalence and abundance in honey bee colonies involved in almond pollination

Ian Cavigli, Katie F. Daughenbaugh, Madison Martin, Michael Lerch, Katie Banner, Emma Garcia, Laura M. Brutscher, Michelle L. Flenniken*

*corresponding author: Michelle L. Flenniken, Department of Plant Sciences and Plant Pathology, Montana State University, Bozeman, MT 59717 USA.

Supplemental Table S1. Primers used in this study.

Genome / Gene Name	NCBI # GI #	Primer Name	Sequence (5'-3')	Product Size (bp)	Reference
**Ribosomal protein L8 (*Apis m.*)**	XM_393671.5<br>GI:571556074	Rpl8Fw<br>Rpl8Rev	TGGATGTTCAACAGGGTTCATA<br>CTGGTGGTGGACGTATTGATAA	121	Evans et al. (2006) Insect Mol Bio
**Lake Sinai virus 1 (LSV1)**	HQ871931<br>GI:335057596	qLSV1-F-2569<br>qLSV1-R-2743**	AGAGGTTGCACGGCAGCATG<br>GGGACGCAGCACGATGCTCA	174	Runckel, Flenniken (2011) PLoS One
**Lake Sinai virus 2 (LSV2)**	HQ888865<br>GI:335057589	qLSV2-F-1722<br>qLSV2-R-1947**	CGTGCTGAGGCCACGGTTGT<br>GCGGTGTCGATCTCGCGGAC	225	Runckel, Flenniken (2011) PLoS One
**Lake Sinai Virus 3 (LSV3)**	JQ480620<br>GI:386289721	LSV3-F-2186<br>LSV3-R-2429	CGTGAGCACGATGAGTCAGT<br>TGGAGGTGCTTGTTGCATAA	243	Daughenbaugh, et al. (2015) Viruses
**Lake Sinai virus 4 (LSV4)**	JX878492<br>GI:512134519	LSV4-F-1896<br>LSV4-R-2278	CCATCTCCTCATCCACGTTT<br>GATTCCCAAATCAGGCTCAA	379	Daughenbaugh, et al. (2015) Viruses
**Lake Sinai virus 5 (LSV5)**	KC880124<br>GI:537367126	LSV5-F-2081<br>LSV5-R-2270	TCCGATACTCACGACGAACA<br>GACACGCGTCAATATCATGG	190	Daughenbaugh, et al. (2015) Viruses
**black queen cell virus (BQCV)**	AF183905<br>GI:8100530	qBQCVorf2F_6664<br>qBQCVorf2R_6805	TCCTCAAATCTGGAGCGAAC<br>GTATTCGCTGGCCGTAAAAC	141	Runckel, Flenniken (2011) PLoS One
**deformed wing virus (DWV)**	AY292384.1<br>GI:31540603	DWVfw1165<br>DWVrev1338	CTTACTCTGCCGTCGCCCA<br>CCGTTAGGAACTCATTATCGCG	173	Chen et a. (2005) J Invert Path
**sacbrood virus (SBV)**	AF092924.1<br>GI:4416206	SBV_F2_5120<br>SBV_R2_5243	AATGTCACCCACGAGTGTTG<br>GCGATGCAACCATACAACTG	123	Daughenbaugh, et al. (2015) Viruses
Crithidia mellificae / Lotmaria passim	PRJNA78249<br>AHIJ01002555<br>Contig 777	qCrFw1<br>qCrRev1	TCCACTCTGCAAACGATGAC<br>GGGCCGAATGGAAAAGATAC	153	Runckel, Flenniken (2011) PLoS One
P. larvae	PRJNA30269	PL2-Fw<br>PL2-Rev	CGGGAGACGCCAGGTTAG<br>TTCTTCCTTGGCAACAGAGC	380	Marinez et al 2010;<br>Marinez et al 2011
**acute bee paralysis virus (ABPV)**	NC_002548.1<br>GI:10314009	qABPV-F-5457<br>qABPV-R-5634	GGATGAGAGAAGACCAATTG<br>CCAATCTTGGGAATAAACATTAGTTC	177	Highfield et al. (2009)<br>Appl Environ Micro;
**chronic bee paralysis virus (CBPV)**	NC_010711.1<br>GI:188543025	CBPV-F_2580<br>CBPV-R_3034	AGTTGTCATGGTTAACAGGATACGAG<br>TCTAATCTTAGCACGAAAGCCGAG	454	Ribiere et al. (2002) Apidologie
**Israeli acute paralysis virus (IAPV)**	NC_009025.1<br>GI:126010924	IAPV_F_7762<br>IAPV_R_7876	GCAGCTATTTTTGGCTGGTC<br>CCAATGTACGCTCATATCG	114	Daughenbaugh, et al. (2015) Viruses
**Kashmir bee virus (KBV)**	NC_004807.1<br>GI:30793779	KBV_F_4470<br>KBV_R_4581	TCGACAAGGACATGATCGAG<br>GAGCCACAAATGGCTTCTTC	111	Stoltz et al. (1995)<br>J Apicult Res
Nosema spp.	DQ235446.1<br>GI:78100222	Nos pan rRNA F-322<br>Nos pan rRNA R-529	GGCAGTTATGGGAAGTAACA<br>GGTCGTCACATTTCATCTCT	207	Chen et al. (2008)<br>J Inv Path
N. ceranae	DQ673615.1<br>GI:110293152	N ceranae F-4186<br>N ceranae R-4435	CGGATAAAAGAGTCCGTTACC<br>TGAGCAGGGTTCTAGGGAT	249	Chen et al. (2008) J Inv Path
M. plutonius	PRJDA73165	MelissoF<br>MelissoR	CAGCTAGTCGGTTTGGTTCC<br>TTGGCTGTAGATAGAATTGACAAT	796	Roetschi et al 2007;<br>Roetschi et al 2008
